# Composition of the intestinal microbiota and its variations between the second and third trimesters in women with gestational diabetes mellitus and without gestational diabetes mellitus

**DOI:** 10.3389/fendo.2023.1126572

**Published:** 2023-07-14

**Authors:** Nana Liu, Yin Sun, Yaxin Wang, Liangkun Ma, Suhan Zhang, Hang Lin

**Affiliations:** Department of Obstetrics and Gynecology, Peking Union Medical College Hospital, Chinese Academy of Medical Sciences & Peking Union Medical College, Beijing, China

**Keywords:** gut microbiota, gestational diabetes mellitus, normal glucose tolerance, healthy pregnant normal glucose tolerance women, second trimester, third trimester

## Abstract

**Objective:**

This study was designed to explore the composition of the intestinal microbiota and its longitudinal variation between the second trimester (T2) and the third trimester (T3) in women with gestational diabetes mellitus (GDM) and pregnant women with normal glucose tolerance.

**Methods:**

This observational study was conducted at Peking Union Medical College Hospital (PUMCH). Women with GDM and pregnant women with normal glucose tolerance were enrolled in the study, and fecal samples were collected during T2 (weeks 24~28) and T3 (weeks 34~38). Fecal samples were analyzed from 49 women with GDM and 42 pregnant women with normal glucose tolerance. The 16S rRNA gene amplicon libraries were sequenced to analyze the microbiota and QIIME2 was used to analyze microbiome bioinformatics.

**Results:**

The four dominant phyla that *Firmicutes*, *Bacteroidetes*, *Actinobacteria* and *Proteobacteria* which accomplish about 99% of the total relative abundance did not significantly change between the T2 and T3 in the GDM and healthy groups. At the genus level, the relative abundance of *Scardovia* (0 vs. 0.25%, P = 0.041) and *Propionibacterium* (0 vs. 0.29%, P = 0.041) increased significantly in the control group, but not in the GDM group. At the phylum level, the relative abundance of *Firmicutes* and *Actinobacteria* was significantly different between women with GDM and pregnant women with normal glucose tolerance in both T2 and T3. In T2 and T3, the relative abundances of *unidentified_Lachnospiraceae*, *Blautia*, and *Parabacteroides* were significantly higher in the GDM group than in the control group (P<0.05). The relative abundance of *Bifidobacterium* in the GDM group was lower than in the control group in both T2 and T3.

**Conclusions:**

The intestinal microbiota composition was stable from T2 to T3 in the GDM and control groups; however, the intestinal microbiota composition was different between the two groups.

## Introduction

The intestinal microbiota, which plays an important role in maintaining human health, colonizes the human intestinal tract (1). In general, the gut microbiota participates in various activities, such as metabolism (2). The gut microbiota can play a role by producing short-chain fatty acids, such as butyrate and propionate (3). The alteration of the intestinal microbiota is associated with many diseases, such as type 2 diabetes and obesity (4–8). Some researchers have recently explored the association between gut microbiota and pregnancy (9–11). The gut microbiota is characterized mainly by an increase in *Actinobacteria* and *Proteobacteria*, with a reduction in the diversity of microbiota and butyrate-producing bacteria during pregnancy (9). Gestational diabetes mellitus (GDM) is a common complication during pregnancy, characterized by the incapability of pancreatic beta cells to respond sufficiently to the increased insulin requirements of pregnancy leading to different degrees of hyperglycemia (12). GDM can pose important short- and long-term health risks for both the mother and the offspring. Although insulin resistance and inflammatory processes have been suggested to be involved in the development of GDM, the specific pathogenesis of GDM remains unclear (13). Therefore, researchers have conducted various studies to explore the gut microbiota characteristics in women with GDM and found differences in the gut microbiota compared with pregnant women with normal glucose tolerance. In women with GDM, opportunistic pathogens in the gut microbiota, such as *Bacteroides* and *Firmicutes* increase, and beneficial bacteria decrease (14).

Various factors, such as dietary intervention and probiotics, influence gut microbiota composition (1). Metabolism can change with the progression of trimesters during pregnancy (15). Koren et al. (9) found that the intestinal microbiota changed dramatically from the first to the third trimester, with a general increase in *Proteobacteria* and *Actinobacteria*, and the microbiota in the third trimester induced greater insulin adiposity than in the first trimester. Abdullah et al. (16) showed that lower α-diversity indices in the GDM group than in the control group, higher abundances in the genera *Acidaminococcus, Clostridium, Megasphaera, and Allisonella*, and lower abundances in *Barnesiella* and *Blautia* but no differences by trimester. Sun et al. (17) found that a decrease in the diversity of intestinal microbial species and changes in the composition of intestinal microbiota with advancing gestation was founded in the control group but not in the GDM group. The gut microbiota in women with GDM may be more stable than that of control group.

To date, the differences in gut microbiota composition between women with GDM and pregnant women with normal glucose tolerance have been explored in various studies, and the conclusions have been similar (18–20). However, a comparison of the intestinal microbiota in women with GDM between different trimesters is lacking. We conducted this prospective observational cohort study to investigate the longitudinal variations of the intestinal microbiota composition from the second (T2) to the third trimester (T3) in women with GDM and pregnant women with normal glucose tolerance.

## Methods

### Ethical approval

This prospective observational cohort study was conducted at the Peking Union Medical College Hospital (PUMCH) between April 2019 and May 2020. This study was reviewed and approved by the Ethics Review Board at PUMCH (approval number HS-1875). Women who met the inclusion criteria and signed an informed consent form were recruited. This study was registered at clinicaltrial.gov (NCT03916354, 04/12/2019). All the procedures were performed in accordance with the Declaration of Helsinki.

### Population and groups

Fifty women with GDM and fifty pregnant women with normal glucose tolerance were enrolled in the study at T2 (24~28 weeks), and basic characteristics such as age, parity, pre-pregnancy body mass index (BMI), height, pre-pregnancy weight and gestational week were collected. Pre-pregnancy BMI was defined as the weight (kg) divided by the square of height (m). The inclusion criteria were as follows: (1) pregnant women, (2) natural pregnancy, (3) singleton pregnancy, and (4) provision of informed consent. Exclusion criteria were: (1) women with pre-pregnancy hypertension, diabetes, and dyslipidemia; (2) severe complications during pregnancy; (3) administration of antibiotics/prebiotics/probiotics during or in the last month before recruitment; (4) any situation of preexisting chronic diseases; and (5) refusal to sign the informed consent.

### Definition

GDM was diagnosed using recommendations of the International Association of the Diabetes and Pregnancy Study Groups (IADPSG), based on the result of a 75 g oral OGTT. Pregnant women who exhibited one or more markers of blood glucose levels higher than the cutoff values (fasting venous plasma glucose levels ≥ 5.1 mmol/L and/or 1 h glucose level ≥ 10.0 mmol/L and/or 2h glucose level ≥ 8.5 mmol/L) were diagnosed with GDM.

### Fecal sample collection

Participants were asked to collect at least 250 mg of feces into a sterile test tube (PSP^®^ Spin Stool DNA Plus Kit) with preservation solution at 24~28 and 34~38weeks. Researchers would instruct the subjects to store the samples in an environment of 4°C and send the samples to hospital within 24 hours. After that, researchers would store the samples at -80°C for DNA extraction.

### Sequencing and analysis of 16S rRNA gene amplicon

DNA was extracted using a QIAamp Fast DNA Stool Mini Kit (Qiagen, Hilden, Germany). The V4 region of the 16S rRNA bacterial gene was amplified by PCR. A TruSeq^®^ DNA PCR-free Sample Preparation Kit was used for library construction and the Illumina NovaSeq 6000 platform was used for sequencing. According to barcode sequence and the PCR amplification primer sequence, each sample data was separated from disembarkation data. After the amputation of barcode and primer sequences using FLASH (V1.2.7, http://ccb.jhu.edu/software/FLASH/) (21) to splice reads of each sample, the splicing sequence for the original tags data (raw tags). Raw tags obtained by splicing need to undergo strict filtering (22) to obtain the high-quality tag data (clean data). According to the QIIME (V1.9.1 http://qiime.org/scripts/split_libraries_fastq.html) (23) tags quality control process, the procedures were as follows: (a) tags to intercept: The raw tags were truncated from the first low-quality base site whose number of consecutive low-quality values (default quality threshold ≤ 19) reached the set length (default length value 3). (b) Tags length filtering: Tags data set obtained by intercepting tags were filtered out tags whose continuous high-quality base length was less than 75% of the length of tags. The tags obtained after the above processing need to be processed to remove the chimeric sequence. The Tags sequence (24) shall be compared with the series annotation database to detect the chimeric sequence, and finally remove the chimeric sequence. Using Uparse software (Uparse v7.0.1001, http://www.drive5.com/uparse/) (25) to cluster all effective tags of all samples. By default, sequences are grouped into operational taxonomic units (OTU) with 97% identification. According to the algorithm principle, the sequences with the highest frequency among OTUs were selected as representative sequences of OTUs. OTU annotation analysis was performed using the Mothur (26) and SSUrRNA databases of SILVA132 (27) (threshold 0.8–1). The Shannon and Simpson indices were calculated using QIIME (version 1.9.1). Beta diversity was calculated using unweighted UniFrac with QIIME. Principal coordinate analysis (PCoA) was performed to obtain the principal coordinates and visualize the complex multidimensional data, and PCoA plots based on unweighted UniFrac distance analysis were used to evaluate beta diversity.

### Statistical analyzes

All statistical analyzes were performed with IBM SPSS 25.0. Clinical baseline characteristics are presented as medians (interquartile range). Continuous variables not normally distributed were reported as medians (interquartile distance), and compared using the Wilcoxon test. The relative abundances of taxa at the phylum and genus levels were compared using the Wilcoxon test. A false discovery rate (FDR)- corrected P < 0.05 was considered statistically significant. All statistical analyzes were performed using two-sided tests.

## Results

### Clinical characteristics of the participants

**Figure 1:**
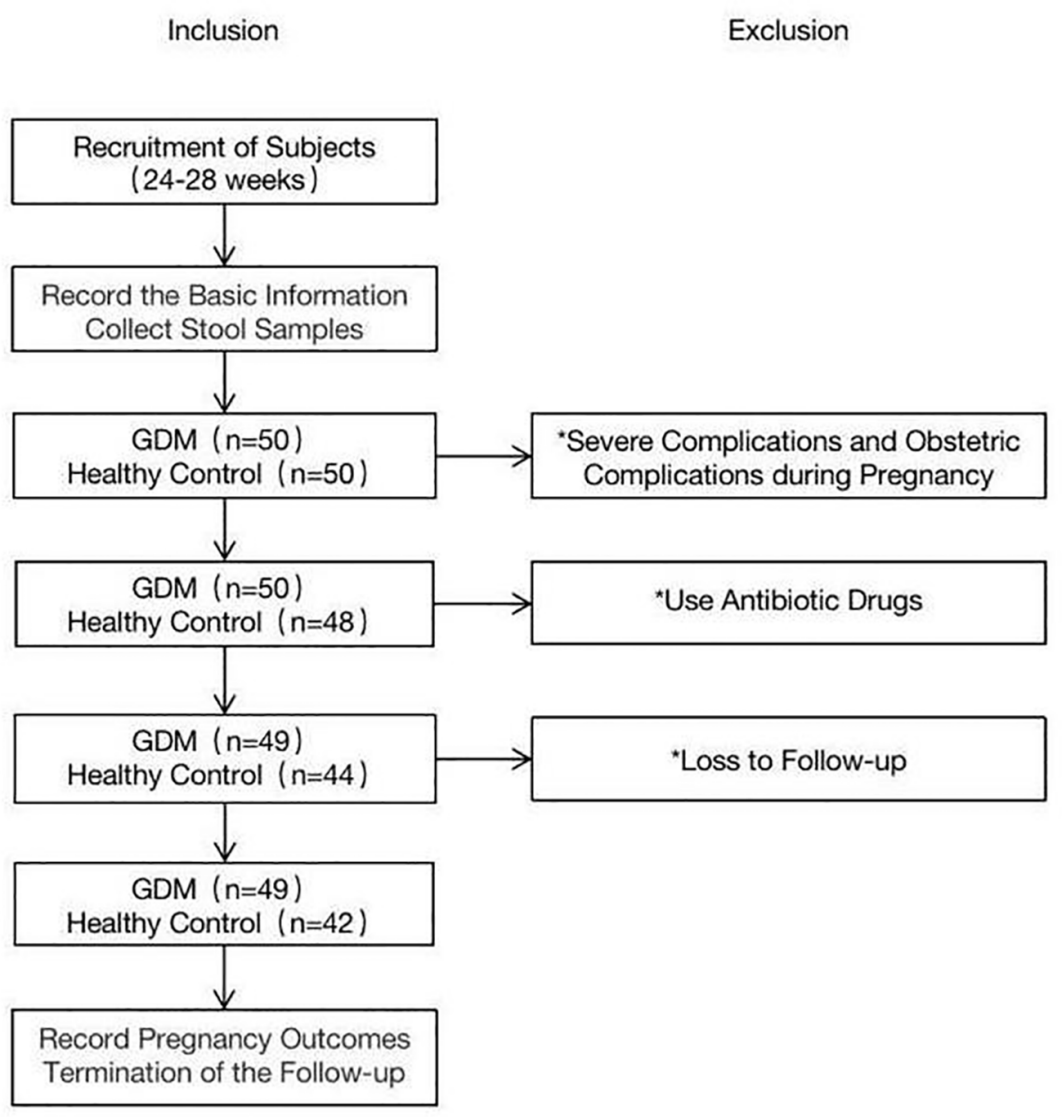
The flow chart of the study. * emphasis on exclusion.

The baseline characteristics of the women with GDM and pregnant women with normal glucose tolerance are summarized in **Table 1**. Fifty women with GDM and fifty controls were enrolled in this study. One person in the GDM group was excluded due to the use of antibiotic drugs. In the control group, two participants were excluded because they experienced serious obstetric complications during pregnancy, four used antibiotic drugs, and two were lost to follow-up (**Figure 1**). The final sample for analyzes included data from 49 women with GDM and 42 pregnant women with normal glucose tolerance. Fecal samples from all participants in the GDM group (n = 49) were collected in T2 (SGDM) and T3 (TGDM). In the control group, one stool sample in T2 and three stool samples in T3 were not received, and eventually 41 and 39 fecal samples were collected in T2 (SHC) and T3 (THC), respectively,.

**Table 1:** Comparison of clinical characteristics in the study groups.

Characteristic	GDM (n=49)	Health women (n=42)	*P* value
Age (year)	33 (32~36.5)	32 (29~34)	0.018*
Parity (number)	1 (1~2)	1 (1~2)	0.438
Pre-pregnancy BMI (kg/m^2^)	22.46 (19.78 ~24.28)	21.05 (19.65~22.68)	0.112
Height(cm)	163.00 (160.00 ~167.00)	163 (162~166.5)	0.592
OGTT-0 hours	4.90 (4.50 ~ 5.25)	4.40 (4.18 ~ 4.60)	< 0.05
OGTT-1 hours	9.80 (8.85 ~ 10.70)	7.55 (6.38 ~8.45)	< 0.05
OGTT-2 hours	8.80 (7.45 ~ 9.40)	6.20 (5.40 ~ 7.23)	< 0.05
Pre-pregnancy Weight (kg)	58 (53.25~64.5)	56.5 (52.5~61.25)	0.217
Gestational week (weeks)	39 (38~39)	39 (38~40)	0.006*

Data presented as median (first quartile, third quartile).

GDM gestational diabetes mellitus, BMI body mass index.

*Statistically significant at P < 0.05.

Women with GDM were more likely to be older (33 (32–36.5) vs. 32 (29–34), P = 0.018) and deliver at lower gestational age (39 (38–39) vs. 39 (38–40), P = 0.006). Other clinical characteristics were not significantly different between the groups (**Table 1**).

### Dynamics in intestinal microbiota in the GDM and control group from T2 to T3

**Figure 2:**
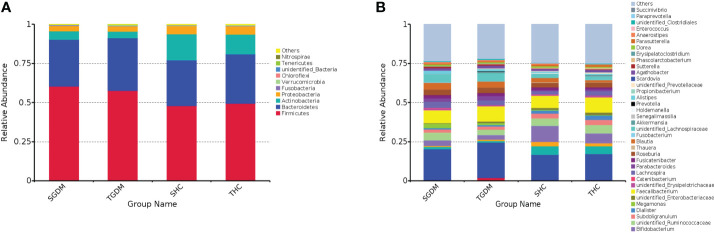
The dynamics in intestinal microbiota in the GDM and control group from T2 to T3 at the phylum and genus level. **(A)** Relative abundance of the top 10 bacterial taxa at the phylum level; **(B)** Relative abundance of the top 40 bacterial taxa at the level of bacterial. GDM, Gestational diabetes mellitus; T2, second trimester; T3, third trimester; SGDM, Second trimester in the GDM group; TGDM, Third trimester in the GDM; SHC, Second trimester in the control group; THC, Third trimester in the control group.

From T2 to T3 in the GDM group, at the phylum level (**Figure 2A**), although not statistically significant, the relative abundances of > 1% of the dominant bacteria, *Firmicutes* (60.31% vs. 57.62%, P = 0.772), *Actinobacteria* (5.43% vs. 4.37%, P = 0.772), and *Proteobacteria* (3.47% vs. 3.27%, P = 0.772), showed a downward trend. *Bacteroides* (29.85% vs. 33.53%, P = 0.772) showed an increasing trend (**Supplement File 1**). The same trend at the genus level (**Figure 2B**), among the top 10 dominant bacteria in the GDM group, although not statistically significant, the relative abundances of *Bacteroides* (20.18% vs. 22.68%, P = 0.791), *Faecalibacterium* (8.25% vs. 9.84%, P = 0.392), *unidentified_Lachnospiraceae* (4.88% vs 5.16%, P = 0.820), *Parabacteroides* (2.28% vs. 2.73%, P = 0.791) showed an increase from T2 to T3, whereas, the relative abundances of *unidentified_Ruminococcaceae* (4.86% vs. 3.54%, P = 0.392), *Blautia* (4.13% vs. 3.83%, P = 0.520), *Roseburia* (3.61% vs. 3.52%, P = 0.791), *Lachnospira* (3.90% vs. 2.89%, P = 0.502), *Bifidobacterium* (3.72% vs. 2.80%, P = 0.392), *Megamonas* (2.81% vs. 1.30%, P = 0.502) showed a decrease from T2 to T3, as illustrated in **Supplement File 2**.

From T2 to T3 in the control group, at the phylum level (**Figure 2A**), although not statistically significant, the relative abundances of *Firmicutes* (47.84% vs. 49.46%, P = 0.969), *Bacteroides* (29.31% vs. 31.44%, P = 0.969), and *Proteobacteria* (5.35% vs. 5.38%, P = 0.915) showed an increasing trend. The relative abundance of *Actinobacteria* (16.59% vs. 12.63%, P = 0.946) showed a downward trend; however, the differences of other bacteria were not statistically significant (**Supplement File 1**). At the genus level (**Figure 2B**), among the top 10 dominant bacteria in the control group, *Bacteroides* (16.31% vs. 16.73%, P = 0.918), *Faecalibacterium* (7.62% vs. 9.70%, P = 0.734), *Bifidobacterium* (10.37% vs. 6.36%, P = 0.637), *Collinsella* (5.52% vs. 5.05%, P = 0.611), *unidentified_Ruminococcaceae* (4.67% vs. 5.09%, P = 0.833), *Subdoligranulum* (3.19% vs. 3.55%, P = 0.611), *Roseburia* (2.90% vs. 3.20%, P = 0.918), *Lachnospira* (2.15% vs. 2.73%, P = 0.918), *Streptococcus* (2.81% vs. 2.02%, P = 0.820), and *unidentified_Lachnospiraceae* (2.54% vs. 2.10%, P = 0.637) (**Supplement File 2**) were both no significant differences from T2 to T3. The relative abundance of *Scardovia* (0 vs. 0.25%, P = 0.041) and *Propionibacterium* (0 vs. 0.29%, P = 0.041) in pregnant women with normal glucose tolerance was significantly higher in T3 than in T2 (**Supplement File 2**).

In T2, at the phylum level (**Figure 2A**), the relative abundance of *Firmicutes* in the GDM group was significantly higher than that in the control group (60.31% vs. 47.84%, P < 0.001), and the relative abundance of *Actinobacteria* in the GDM group was significantly lower than that in the control group (5.43% vs. 16.59%, P = 0.009). The abundance of other bacteria is described in **Supplement File 1**. At the genus level (**Figure 2B**), the relative abundances of *unidentified_Lachnospiraceae*e (4.88% vs. 2.55%, P < 0.001), *Roseburia* (3.61% vs. 2.90%, P = 0.041), *Lachnospira* (3.90% vs. 2.15%, P = 0.004), *Blautia* (4.13% vs. 2.76%, P = 0), and *Parabacteroides* (2.27% vs. 0.73%, P = 0) in the GDM group were higher than those in the control group. The relative abundance of *Bifidobacterium* in the GDM group was lower than that in the control group (3.72% vs. 10.37%, P = 0.012). The relative abundances of other bacteria were lower in the GDM group than in the control group (**Supplement File 2**).

In T3, at the phylum level (**Figure 2A**), the relative abundance of *Firmicutes* (57.62% vs. 49.46%, P = 0.044) in the GDM group was significantly higher than that in the control group. The relative abundance of *Actinobacteria* (4.37% vs. 12.63%, P = 0.007) in the GDM group was significantly lower than in the control group. The relative abundances of other bacteria are detailed in **Supplement File 1**. At the genus level (**Figure 2B**), the relative abundances of *unidentified_Lachnospiraceae* (5.16% vs. 2.11%, P = 0), *Blautia* (3.83% vs.1.46%, P = 0), *Parabacteroides* (2.73% vs. 1.18%, P = 0), and *Megamonas* (1.31% vs. 0.21%, P = 0.038) in the GDM group were significantly higher than those in the control group. The relative abundance of *Bifidobacterium* (2.80% vs. 6.36%, P = 0.022) in the GDM group was significantly lower than that in the control group (**Supplement file 2**). The relative abundances of other bacteria are detailed in **Supplement File 2**.

### OTUs

**Figure 3:**
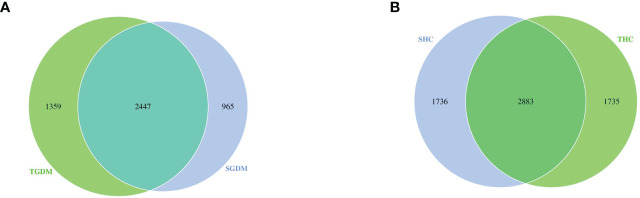
Venn diagram among the GDM and control groups. **(A)** The overlaps of OTUs in the GDM group. **(B)** The overlaps of OTUs in the control groups. GDM, Gestational diabetes mellitus; SGDM, Second trimester in the GDM group; TGDM, Third trimester in the GDM group; SHC, Second trimester in the control group; THC, Third trimester in the control group.

Venn diagrams were drawn on the basis of the number of OTUs of samples in the GDM and control groups (**Figure 3**). As shown in the figure, in the GDM group, the total number of OTUs in T2 and T3 was 3412 and 3806, respectively. The number of common OTUs in T2 and T3 was 2447; the number of unique OTUs in T2 and T3 was 965 and 1359, respectively (**Figure 3A**). The number of unique OTUs in T2 represented 28.28% of the total OTUs in T2 and 35.71% of the total OTUs in T3. In the control group, the total number of OTUs in T2 and T3 was 4619 and 4618, respectively. The number of common OTUs in T2 and T3 was 2883, and the unique numbers of OTUs in T2 and T3 were 1736 and 1735, respectively (**Figure 3B**). Unique OTUs in T2 accounted for 37.58% of the total OTUs in T2 and 37.57% of the total OTUs in T3.

### The alpha and beta diversity

**Figure 4:**
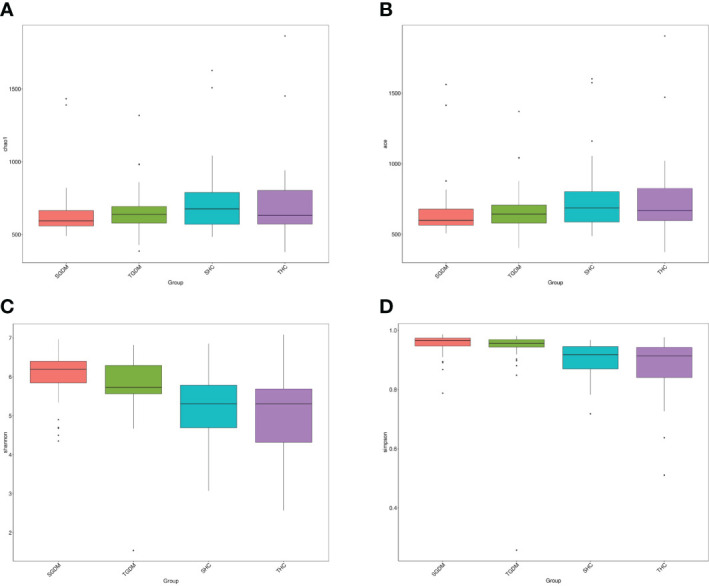
The alpha diversity of intestinal microbiota in the GDM and control groups. **(A)** Chao1 estimator, **(B)** abundance-based coverage estimator (ACE), **(C)** Shannon, **(D)** Simpson. GDM, Gestational diabetes mellitus. SGDM: Second trimester in the GDM group; TGDM, Third trimester in the GDM group; SHC, Second trimester in the control group; THC, Third trimester in the control group.

In the GDM group, there was no significant differences in the Chao index (P=0.123) (**Figure 4A**) and ACE index (P=0.201) (**Figure 4B**) were observed from T2 to T3. The same trend in the control group. In the GDM group, there was no significant difference in changes in the Shannon index (**Figure 4C**) (6.039 vs 5.822, P = 0.078) and the Simpson index (**Figure 4D**) was observed from T2 to T3 (0.953 vs 0.937, P = 0.177). The Shannon index (**Figure 4C**) (5.188 vs. 5.043, P=0.795) and the Simpson index (**Figure 4D**) (0.904 vs. 0.880, P = 0.824) in the control group from T2 to T3 were not statistically significant. The Shannon index in T2 (6.039 vs 5.188, P = 0) and T3 (5.822 vs 5.043, P = 0) in the GDM group were both significantly higher than those in the control group, and the Simpson index in T2 (0.953 vs. 0.904, P < 0.001) and T3 (0.937 vs. 0.880, P <.001) in the GDM group were both significantly higher than those in the control group.

**Figure 5:**
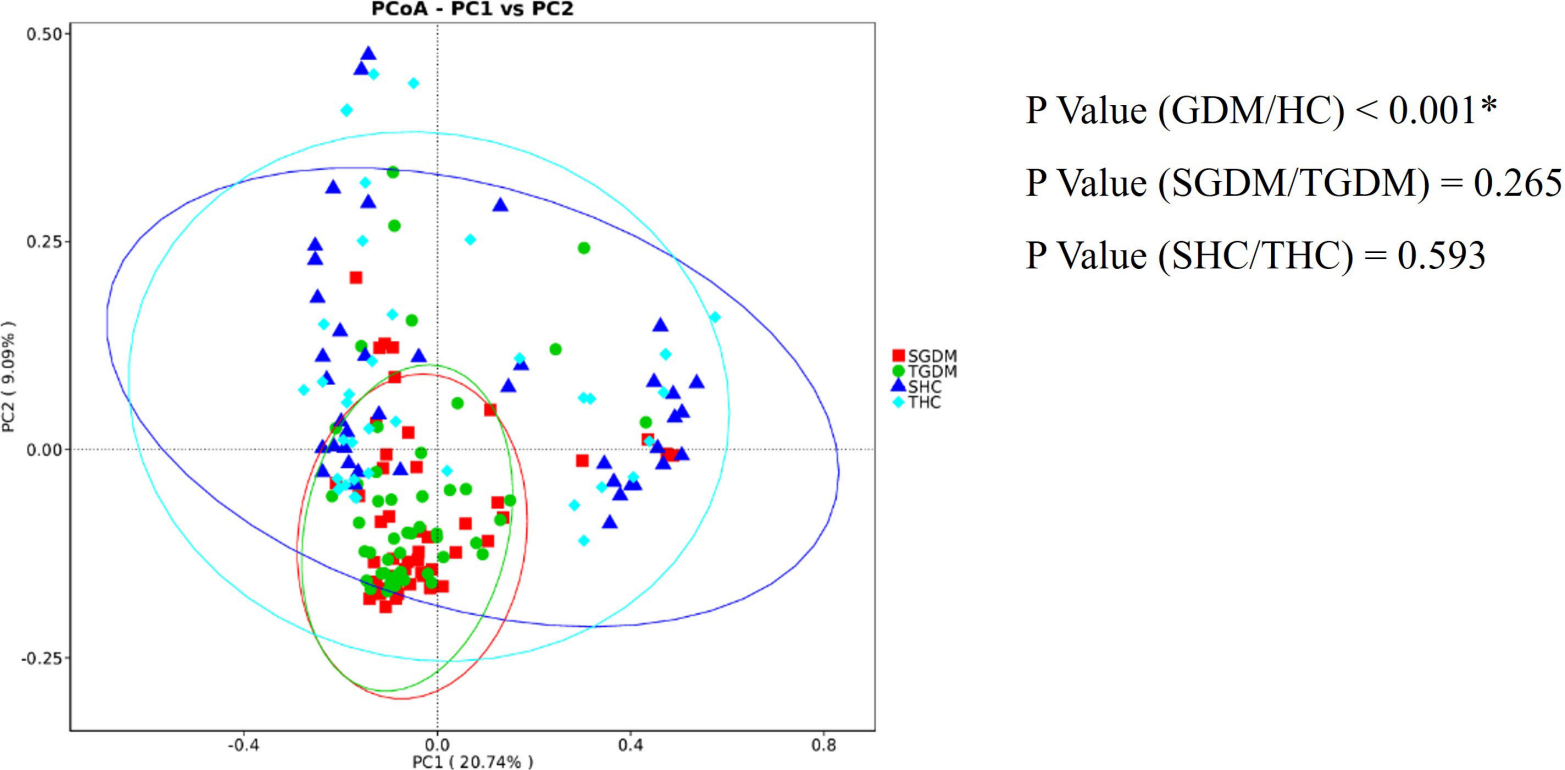
PCoA shows the dispersal of gut microbiota between trimesters in the GDM and healthy control groups. Red represents GDM samples in T2, green represents GDM samples in T3, dark blue represents samples of the control group in T2, and light blue represents samples of the control group in T3. SGDM, GDM group in the second trimester; TGDM, GDM group in the third trimester; SHC, control group in the second trimester; THC, control group in the third trimester; PCoA, Principal Coordinate Analysis. *P value < 0.05.

PC1 was the main coordinate component that caused the largest difference in the samples, with an explanatory degree of 20.74%, followed by PC2, with an explanatory degree of 9.09% (**Figure 5**). According to the AMOVA analysis, there were no significant differences in the gut composition microbiota in T2 and T3 in the GDM (P = 0.265) and control groups (P = 0.593). However, there was a significant difference in the composition of the gut microbiota between the GDM and control groups (P < 0.001). The distribution of the intestinal microbiota in T2 and T3 was similar in the GDM and control groups; however, the distribution distance of the GDM group was relatively far compared to that of the control group.

## Discussion

This study explored the composition of the intestinal microbiota and its alternative characteristics from T2 to T3 in women with GDM and pregnant women with normal glucose tolerance. The results showed that *Scardovia* and *Propionibacterium* were significantly higher in T3 than in T2 in the control group, but not in the GDM group. The changes in the relative abundance of the remaining bacteria from T2 to T3 were stable in the GDM and control groups. Nevertheless, there were significant differences in the composition of the gut microbiota in the GDM and control groups in both T2 and T3.

We found that the dominant bacteria were composed of four phyla: *Firmicutes, Bacteroidetes, Proteobacteria*, and *Actinobacteria* at the phylum level in both the GDM and the control groups, which was consistent with the results of Tang et al. (28). Ma et al. (29) found that the four dominant phyla were *Firmicutes*, *Bacteroidetes, Proteobacteria*, and *Tenericutes*. *Actinobacteria* (30) act as markers of GDM and are positively correlated with fasting blood glucose levels; however, this association was not present after adjusting for pre-pregnancy body mass index (BMI). *Tenericutes* (31) are the dominant bacteria in the neonatal oral microbiota in babies of women with GDM. In our study, the relative abundance of *Tenericutes* was less than 1%, which may be explained by the type of samples studied and sample size. At the genus level, *Bacteroides, Faecalibacterium, unidentified_Lachnospiraceae*, *unidentified_Ruminococcaceae, Roseburia*, *Lachnospira*, and *Bifidobacterium* were the dominant bacteria in both the GDM and control groups. *Blautia, Parabacteroides*, and *Megamonas* were the dominant bacteria in the GDM group, while *Collinsella, Subdoligranulum*, and *Streptococcus* were the dominant bacteria in the control group.These GDM-enriched genus may participate in the development of GDM by influencing host immune status. *Blautia* (32), which is significantly associated with host dysfunctions, such as obesity, diabetes, and various inflammatory diseases, is a genus of biotransformative bacteria with probiotic properties that can regulate host health and alleviate metabolic syndrome. *Parabacteroides* are enriched in overweight women (30) and in women with GDM (18), which is consistent with our findings. *Megamonas* is enriched in obese women (16, 18) and has a positive relationship with glucose tolerance (18). *Megamonas* was the dominant bacterium specific to women with GDM; however, women with GDM were not classified by pre-pregnancy weight class in our study. Women with a history of GDM have a high abundance of *Collinsella* in their postpartum gut microbiota, and *Collinsella* has the potential to be a marker for the future development of type 2 diabetes in women with a history of GDM (30). However, previous studies have reported that *Collinsella* increases in healthy pregnancies (33, 34). In the present study, the relative abundance of *Collinsella* was higher in the control group. This difference may need the studies that enroll more subjects to explain. *Subdoligranulum*, which produces short-chain fatty acids such as butyrate, is negatively associated with human fat accumulation, insulin resistance, insulin, CRP, IL6, and other markers (35). A study (36) found that the relative abundance of *Streptococcus* in overweight, obese, and diabetic patients was lower than that of healthy controls, and Hajifaraji et al. (37) found that the combination of *Streptococcus* with other probiotics had a positive outcome in the treatment of metabolic diseases.

In our study, we found that the composition of the intestinal microbiota in the GDM and pregnant women with normal glucose tolerance was relatively stable from T2 to T3. Only the relative abundance of *Scardovia* and *Propionibacterium* in T3 was significantly higher than in T2 in pregnant women with normal glucose tolerance. Members of *Scardovia* are one of the seven genera of the *Bifidobacteriaceae* family and recognized as the healthy gut microbiota (38). *Scardovia* can produce acetic acid from glucose, together with small amounts of lactic and formic acid (39). It is reported that *Propionibacterium* can ameliorate insulin resistance by obesity (40).Insulin resistance, which is emphasized in the development of GDM in the late pregnancy, is associated with a reduced abundance of butyrate-producing bacteria (41–43). Ferrocino et al. found that an increase in *Firmicutes* and a reduction in *Bacteroidetes* and *Actinobacteria* from T2 to T3 in women with GDM who adhered to dietary recommendations showed a better metabolic and inflammatory pattern at the end of the study and a clear decrease in *Bacteroidetes* (44). We found that at the phylum level, the *Firmicutes*/*Bacteroidetes* ratio both decreased in the GDM group and control group from T2 to T3. The increased *Firmicutes*/*Bacteroidetes* ratio is associated with obesity and inflammation (45), and the decreased *Firmicutes*/*Bacteroidetes* ratio in our study may be related to factors such as dietary modifications. However, Sun et al. (17) found a phenomenon that with advancing gestation, decreasing trends in the *Firmicutes*/*Bacteroides* ratio were observed in the control group but not in the GDM group. In addition, they also found that time-dependent alterations in gut microbiota composition were found in the control group but not in the GDM group. Compared to women with normal glucose, women with GDM tended to have a reduced intestinal microbiota diversity in the first trimester, while differences in intestinal microbiota composition were consistent in T2 and T3. Our research does not observe the composition of the gut microbiota in the first trimester and our study also observed the stable composition of the gut microbial in T2 and T3 in women with GDM. Women who develop GDM may have alterations in intestinal microbial composition from early pregnancy, explained by metabolic status. *Bacteroides*, a Gram-negative bacterium, can produce large amounts of LPS, leading to inflammation. LPS mainly activates inflammation via the Toll-like receptor 4 signaling pathway (46). From the first to the third trimester, women gain adiposity and have higher circulating levels of insulin (9). In women with GDM, two main inflammatory pathways, nuclear factor kappa B (NF-kB) and signal transducers and activators of the transcription 3 (STAT3) pathways, have been identified (13). The findings of this study provide evidence to explain the stable status of GDM.

In this study, the Shannon and Simpson indices of the GDM and healthy pregnancy groups both decreased from T2 to T3; however, the Shannon and Simpson indices of the GDM women were significantly higher than those of pregnant women with normal glucose tolerance. Our study was consistent with previous researches, showing the decreased microbial diversity with advancing gestation (9). This phenomenon might be due to the metabolic modifications occurring pregnancy, including changes of blood glucose and hormone. Higher α diversity values were associated with a lower incidence of type 2 diabetes, which was not affected by energy intake, exercise, education, smoking, or medication (47). Insulin resistance and elevated blood glucose levels can increase the risk of type 2 diabetes (48). With increasing gestational age, the level of insulin resistance increases to meet the nutritional supply of the mother and child (49). A lower Shannon index significantly correlated with blood glucose levels in patients with diabetes (19). The high Shannon and Simpson indices of the GDM group in this study could be explained by the inherent differences between the GDM and control groups. According to previous studies, β diversity is associated with insulin resistance and plasma OGTT levels (19, 47). Different methods to investigate beta diversity can influence the results. Unweighted UniFrac is sensitive to the absence and presence of low abundant bacteria, while both weighted Unifrac and Bray Cruits are more sensitive to the more abundant bacteria. In our study, unweighted Unifrac is used to investigate beta diversity. More methods should be used to claim beta diversity.

Our study explored the alterations of gut microbiota with the increasing gestational age in women with GDM and pregnant women with normal glucose tolerance. So far, few studies have explored the changes of intestinal microbiota composition in women with GDM during different trimesters. The longitudinal study will contribute to the understanding of the association between gut microbiota and GDM and provide the thinking way to predict the occurrence of GDM during early pregnancy. There are also some limitations in our study. First, this study was conducted at a single center with a limited sample size, and larger studies are needed in the future to verify the results of the study. Second, our study is an observational study and data may lack causality. There need more randomized control tests to research the association of dynamic gut microbiota composition between different trimesters in women with GDM. Third, lifestyle management is the first-line treatment for GDM but the diet patterns of the participants in this study were lack.

## Conclusion

Our study indicated that the composition of the gut microbiota was stable with advancing gestation in women with GDM compared with the control group and gut microbiota composition was obviously different between women with GDM and controls. These findings may help explore the etiology of GDM from new perspective of the relationship between gut microbiota and glucose metabolism.

## Data availability statement

The original contributions presented in the study are publicly available. This data can be found here: [https://www.ncbi.nlm.nih.gov//PRJNA937449].

## Ethics statement

The studies involving human participants were reviewed and approved by Peking Union Medical College Hospital (PUMCH). The patients/participants provided their written informed consent to participate in this study.

## Author contributions

Study design: YS and LM. Sample and data collection: SZ and HL. Analysis and interpretation of data: NL and YW. Drafting the manuscript: NL. Critical revision of the manuscript for important intellectual content: YS. Statistical analysis: NL and YW. Obtained funding: LM. Study Supervision: YS and LM. All authors contributed to the article and approved the submitted version.

## References

[1] Fan Y Pedersen O. Gut microbiota in human metabolic health and disease. Nat Rev Microbiol (2021) 19(1):55–71. doi: 10.1038/s41579-020-0433-9 32887946

[2] Claus SP Ellero SL Berger B Krause L Bruttin A Molina J. Colonization-induced host-gut microbial metabolic interaction. mBio (2011) 2(2):e00271–00210. doi: 10.1128/mBio.00271-10 PMC3045766 21363910

[3] Clarke G Stilling RM Kennedy PJ Stanton C Cryan JF Dinan TG. Minireview: gut microbiota: the neglected endocrine organ. Mol Endocrinol (Baltimore Md) (2014) 28(8):1221–38. doi: 10.1210/me.2014-1108 PMC5414803 24892638

[4] Meijnikman AS Gerdes VE Nieuwdorp M Herrema H. Evaluating causality of gut microbiota in obesity and diabetes in humans. Endocrine Rev (2018) 39(2):133–53. doi: 10.1210/er.2017-00192 29309555

[5] Martínez-Cuesta MC Del Campo R Garriga-García M Peláez C Requena T. Taxonomic characterization and short-chain fatty acids production of the obese microbiota. Front Cell infection Microbiol (2021) 11:598093. doi: 10.3389/fcimb.2021.598093 PMC8242951 34222034

[6] Li H Fang Q Nie Q Hu J Yang C Huang T. Hypoglycemic and hypolipidemic mechanism of tea polysaccharides on type 2 diabetic rats via gut microbiota and metabolism alteration. J Agric Food Chem (2020) 68(37):10015–28. doi: 10.1021/acs.jafc.0c01968 32811143

[7] Ahmad A Yang W Chen G Shafiq M Javed S Ali Zaidi SS. Analysis of gut microbiota of obese individuals with type 2 diabetes and healthy individuals. PloS One (2019) 14(12):e0226372. doi: 10.1371/journal.pone.0226372 31891582 PMC6938335

[8] Larsen N Vogensen FK van den Berg FW Nielsen DS Andreasen AS Pedersen BK. Gut microbiota in human adults with type 2 diabetes differs from non-diabetic adults. PloS One (2010) 5(2):e9085. doi: 10.1371/journal.pone.0009085 20140211 PMC2816710

[9] Koren O Goodrich JK Cullender TC Spor A Laitinen K Bäckhed HK. Host remodeling of the gut microbiome and metabolic changes during pregnancy. Cell (2012) 150(3):470–80. doi: 10.1016/j.cell.2012.07.008 PMC3505857 22863002

[10] Collado MC Isolauri E Laitinen K Salminen S. Distinct composition of gut microbiota during pregnancy in overweight and normal-weight women. Am J Clin Nutr (2008) 88(4):894–9. doi: 10.1093/ajcn/88.4.894 18842773

[11] DiGiulio DB Callahan BJ McMurdie PJ Costello EK Lyell DJ Robaczewska A. Temporal and spatial variation of the human microbiota during pregnancy. Proc Natl Acad Sci United States America (2015) 112(35):11060–5. doi: 10.1073/pnas.1502875112 PMC4568272 26283357

[12] Johns EC Denison FC Norman JE Reynolds RM. Gestational diabetes mellitus: mechanisms, treatment, and complications. Trends Endocrinol metabolism: TEM (2018) 29(11):743–54. doi: 10.1016/j.tem.2018.09.004 30297319

[13] de Mendonça E Fragoso MBT de Oliveira JM Xavier JA Goulart MOF de Oliveira ACM. Gestational diabetes mellitus: the crosslink among inflammation, nitroxidative stress, intestinal microbiota and alternative therapies. Antioxidants (Basel Switzerland) (2022) 11(1):129. doi: 10.3390/antiox11010129 35052633 PMC8773111

[14] Medici Dualib P Ogassavara J Mattar R Mariko Koga da Silva E Atala Dib S de Almeida Pititto B. Gut microbiota and gestational diabetes mellitus: a systematic review. Diabetes Res Clin Pract (2021) 180:109078. doi: 10.1016/j.diabres.2021.109078 34599971

[15] Liang L Rasmussen MH Piening B Shen X Chen S Röst H. Metabolic dynamics and prediction of gestational age and time to delivery in pregnant women. Cell (2020) 181(7):1680–1692.e1615. doi: 10.1016/j.cell.2020.05.002 32589958 PMC7327522

[16] Abdullah B Daud S Aazmi MS Idorus MY Mahamooth MIJ. Gut microbiota in pregnant Malaysian women: a comparison between trimesters, body mass index and gestational diabetes status. BMC pregnancy childbirth (2022) 22(1):152. doi: 10.1186/s12884-022-04472-x 35209853 PMC8876553

[17] Sun Z Pan XF Li X Jiang L Hu P Wang Y. The gut microbiome dynamically associates with host glucose metabolism throughout pregnancy: longitudinal findings from a matched case-control study of gestational diabetes mellitus. Adv Sci (Weinh) (2023) 2023:e2205289. doi: 10.1002/advs.202205289 PMC10074094 36683149

[18] Kuang YS Lu JH Li SH Li JH Yuan MY He JR. Connections between the human gut microbiome and gestational diabetes mellitus. GigaScience (2017) 6(8):1–12. doi: 10.1093/gigascience/gix058 PMC5597849 28873967

[19] Chen T Zhang Y Zhang Y Shan C Zhang Y Fang K. Relationships between gut microbiota, plasma glucose and gestational diabetes mellitus. J Diabetes Invest (2021) 12(4):641–50. doi: 10.1111/jdi.13373 PMC8015828 32702151

[20] Ye G Zhang L Wang M Chen Y Gu S Wang K. The gut microbiota in women suffering from gestational diabetes mellitus with the failure of glycemic control by lifestyle modification. J Diabetes Res (2019) 2019:6081248. doi: 10.1155/2019/6081248 31772944 PMC6854930

[21] Magoč T Salzberg SL. FLASH: fast length adjustment of short reads to improve genome assemblies. Bioinf (Oxford England) (2011) 27(21):2957–63. doi: 10.1093/bioinformatics/btr507 PMC3198573 21903629

[22] Bokulich NA Subramanian S Faith JJ Gevers D Gordon JI Knight R. Quality-filtering vastly improves diversity estimates from illumina amplicon sequencing. Nat Methods (2013) 10(1):57–9. doi: 10.1038/nmeth.2276 PMC3531572 23202435

[23] Caporaso JG Kuczynski J Stombaugh J Bittinger K Bushman FD Costello EK. QIIME allows analysis of high-throughput community sequencing data. Nat Methods (2010) 7(5):335–6. doi: 10.1038/nmeth.f.303 PMC3156573 20383131

[24] Rognes T Flouri T Nichols B Quince C Mahé F. VSEARCH: a versatile open source tool for metagenomics. PeerJ (2016) 4:e2584. doi: 10.7717/peerj.2584 27781170 PMC5075697

[25] Haas BJ Gevers D Earl AM Feldgarden M Ward DV Giannoukos G. Chimeric 16S rRNA sequence formation and detection in Sanger and 454-pyrosequenced PCR amplicons. Genome Res (2011) 21(3):494–504. doi: 10.1101/gr.112730.110 21212162 PMC3044863

[26] Edgar RC. UPARSE: highly accurate OTU sequences from microbial amplicon reads. Nat Methods (2013) 10(10):996–8. doi: 10.1038/nmeth.2604 23955772

[27] Quast C Pruesse E Yilmaz P Gerken J Schweer T Yarza P. The SILVA ribosomal RNA gene database project: improved data processing and web-based tools. Nucleic Acids Res (2013) 41(Database issue):D590–596. doi: 10.1093/nar/gks1219 PMC3531112 23193283

[28] Tang N Luo ZC Zhang L Zheng T Fan P Tao Y. The association between gestational diabetes and microbiota in placenta and cord blood. Front Endocrinol (2020) 11:550319. doi: 10.3389/fendo.2020.550319 PMC7609904 33193081

[29] Ma S You Y Huang L Long S Zhang J Guo C. Alterations in gut microbiota of gestational diabetes patients during the first trimester of pregnancy. Front Cell infection Microbiol (2020) 10:58. doi: 10.3389/fcimb.2020.00058 PMC7056672 32175285

[30] Crusell MKW Hansen TH Nielsen T Allin KH Rühlemann MC Damm P. Gestational diabetes is associated with change in the gut microbiota composition in third trimester of pregnancy and postpartum. Microbiome (2018) 6(1):89. doi: 10.1186/s40168-018-0472-x 29764499 PMC5952429

[31] He Z Wu J Xiao B Xiao S Li H Wu K. The initial oral microbiota of neonates among subjects with gestational diabetes mellitus. Front Pediatr (2019) 7:513. doi: 10.3389/fped.2019.00513 31921726 PMC6914726

[32] Liu X Mao B Gu J Wu J Cui S Wang G. Blautia-a new functional genus with potential probiotic properties? Gut Microbes (2021) 13(1):1–21. doi: 10.1080/19490976.2021.1875796 PMC7872077 33525961

[33] Zheng W Xu Q Huang W Yan Q Chen Y Zhang L. Gestational diabetes mellitus is associated with reduced dynamics of gut microbiota during the first half of pregnancy. mSystems (2020) 5(2):e00109-20. doi: 10.1128/mSystems.00109-20 32209715 PMC7093821

[34] Nuriel-Ohayon M Neuman H Ziv O Belogolovski A Barsheshet Y Bloch N. Progesterone increases bifidobacterium relative abundance during late pregnancy. Cell Rep (2019) 27(3):730–736.e733. doi: 10.1016/j.celrep.2019.03.075 30995472

[35] Van Hul M Le Roy T Prifti E Dao MC Paquot A Zucker JD. From correlation to causality: the case of subdoligranulum. Gut Microbes (2020) 12(1):1–13. doi: 10.1080/19490976.2020.1849998 PMC7744154 33323004

[36] Ma M Su J Wang Y Wang L Li Y Ding G. Association of body mass index and intestinal (faecal) streptococcus in adults in xining city, China P.R. Benef Microbes (2022) 13(6):1–8. doi: 10.3920/BM2021.0046 36264094

[37] Hajifaraji M Jahanjou F Abbasalizadeh F Aghamohammadzadeh N Abbasi MM Dolatkhah N. Effect of probiotic supplements in women with gestational diabetes mellitus on inflammation and oxidative stress biomarkers: a randomized clinical trial. Asia Pacific J Clin Nutr (2018) 27(3):581–91. doi: 10.6133/apjcn.082017.03 29737805

[38] Kameda M Abiko Y Washio J Tanner ACR Kressirer CA Mizoguchi I. Sugar metabolism of scardovia wiggsiae, a novel caries-associated bacterium. Front Microbiol (2020) 11:479. doi: 10.3389/fmicb.2020.00479 32269556 PMC7109253

[39] Manome A Abiko Y Kawashima J Washio J Fukumoto S Takahashi N. Acidogenic potential of oral bifidobacterium and its high fluoride tolerance. Front Microbiol (2019) 10:1099. doi: 10.3389/fmicb.2019.01099 31156604 PMC6532017

[40] An M Park YH Lim YH. Antiobesity and antidiabetic effects of the dairy bacterium propionibacterium freudenreichii MJ2 in high-fat diet-induced obese mice by modulating lipid metabolism. Sci Rep (2021) 11(1):2481. doi: 10.1038/s41598-021-82282-5 33510408 PMC7844274

[41] American Diabetes Association. (2015).(2) classification and diagnosis of diabetes. Diabetes Care (2015) 38 (Suppl):S8–s16. doi: 10.2337/dc15-S005 25537714

[42] Crommen S Simon MC. Microbial regulation of glucose metabolism and insulin resistance. Genes (Basel) (2017) 9(1):10. doi: 10.3390/genes9010010 29286343 PMC5793163

[43] Serino M Fernández-Real JM García-Fuentes E Queipo-Ortuño M Moreno-Navarrete JM Sánchez A. The gut microbiota profile is associated with insulin action in humans. Acta diabetologica (2013) 50(5):753–61. doi: 10.1007/s00592-012-0410-5 PMC3898146 22711164

[44] Ferrocino I Ponzo V Gambino R Zarovska A Leone F Monzeglio C. Changes in the gut microbiota composition during pregnancy in patients with gestational diabetes mellitus (GDM). Sci Rep (2018) 8(1):12216. doi: 10.1038/s41598-018-30735-9 30111822 PMC6093919

[45] Roselli M Devirgiliis C Zinno P Guantario B Finamore A Rami R. Impact of supplementation with a food-derived microbial community on obesity-associated inflammation and gut microbiota composition. Genes Nutr (2017) 12:25. doi: 10.1186/s12263-017-0583-1 29043005 PMC5628415

[46] Liu Y Qin S Feng Y Song Y Lv N Liu F. Perturbations of gut microbiota in gestational diabetes mellitus patients induce hyperglycemia in germ-free mice. J Dev origins Health Dis (2020) 11(6):580–8. doi: 10.1017/S2040174420000768 32924908

[47] Chen Z Radjabzadeh D Chen L Kurilshikov A Kavousi M Ahmadizar F. Association of insulin resistance and type 2 diabetes with gut microbial diversity: a microbiome-wide analysis from population studies. JAMA network Open (2021) 4(7):e2118811. doi: 10.1001/jamanetworkopen.2021.18811 34323983 PMC8322996

[48] Kolb H Kempf K Röhling M Martin S. Insulin: too much of a good thing is bad. BMC Med (2020) 18(1):224. doi: 10.1186/s12916-020-01688-6 32819363 PMC7441661

[49] Barbour LA McCurdy CE Hernandez TL Kirwan JP Catalano PM Friedman JE. Cellular mechanisms for insulin resistance in normal pregnancy and gestational diabetes. Diabetes Care (2007) 30 Suppl 2:S112–119. doi: 10.2337/dc07-s202 17596458

